# Genome-Wide Identification and Expression Analysis of bHLH-MYC Family Genes from Mustard That May Be Important in Trichome Formation

**DOI:** 10.3390/plants14020268

**Published:** 2025-01-18

**Authors:** Jianzhong Li, Guoliang Li, Caishuo Zhu, Shaoxing Wang, Shifan Zhang, Fei Li, Hui Zhang, Rifei Sun, Lingyun Yuan, Guohu Chen, Xiaoyan Tang, Chenggang Wang, Shujiang Zhang

**Affiliations:** 1 College of Horticulture, Anhui Agricultural University, Hefei 230036, China; 18335863430@163.com (J.L.);; 2 State Key Laboratory of Vegetable Biobreeding, Institute of Vegetables and Flowers, Chinese Academy of Agricultural Sciences, Beijing 100081, China; liguoliang@caas.cn (G.L.);; 3 College of Horticulture, Shanxi Agricultural University, Jinzhong 030801, China

**Keywords:** mustard, leaf trichome, MBW, bHLH, MYC

## Abstract

The trichomes of mustard leaves have significance due to their ability to combat unfavorable external conditions and enhance disease resistance. It was demonstrated that the MYB-bHLH-WD40 (MBW) ternary complex consists of MYB, basic Helix-Loop-Helix (bHLH), and WD40-repeat (WD40) family proteins and plays a key role in regulating trichome formation and density. The bHLH gene family, particularly the Myelocytomatosis (MYC) proteins that possess the structural bHLH domain (termed bHLH-MYC), are crucial to the formation and development of leaf trichomes in plants. bHLH constitutes one of the largest families of transcription factors in eukaryotes, of which MYC is a subfamily member. However, studies on bHLH-MYC transcription factors in mustard have yet to be reported. In this study, a total of 45 bHLH-MYC transcription factors were identified within the *Brassica juncea* genome, and a comprehensive series of bioinformatic analyses were conducted on their structures and properties: an examination of protein physicochemical properties, an exploration of conserved structural domains, an assessment of chromosomal positional distributions, an analysis of the conserved motifs, an evaluation of the gene structures, microsynteny analyses, three-dimensional structure prediction, and an analysis of sequence signatures. Finally, transcriptome analyses and a subcellular localization examination were performed. The results revealed that these transcription factors were unevenly distributed across 18 chromosomes, showing relatively consistent conserved motifs and gene structures and high homology. The final results of the transcriptome analysis and gene annotation showed a high degree of variability in the expression of bHLH-MYC transcription factors. Five genes that may be associated with trichome development (*BjuVA09G22490*, *BjuVA09G13750*, *BjuVB04G14560*, *BjuVA05G24810*, and *BjuVA06G44820*) were identified. The subcellular localization results indicated that the transcription and translation products of these five genes were expressed in the same organelle: the nucleus. This finding provides a basis for elucidating the roles of bHLH-MYC family members in plant growth and development, and the molecular mechanisms underlying trichome development in mustard leaves.

## 1. Introduction

Mustard (*Brassica juncea* (*L*). *Czern*.) is a heterotetraploid plant (AABB, 2n = 36) belonging to the genus Brassica in the family Cruciferae. It is cultivated globally as both an oilseed and a vegetable [1]. According to the knoema website, the world production of mustard in 2021 was 532,769.12 tons on 631,695 hectares, with a yield of about 8434 kg/ha. Asia produced 282,720.55 tons in the same year, accounting for 53% of world production. China is one of the primary production regions for mustard. The northwest region of China is the primary production center for Chinese mustard, while the Sichuan Basin is a secondary production center for mustard [2]. As one of the characteristic vegetables of China, mustard contains a wide range of vitamins and minerals that enrich the palate while having secondary metabolites such as flavonoids, polyphenols, and sulfur-containing compounds [3]. They can have beneficial health-related effects on the human body by enhancing the immune system, preventing cancer, and inhibiting Reactive Oxygen Species (ROS) with high antioxidant activity [4,5]. According to its edible parts, it can be classified as leaf mustard, stem mustard, and root mustard. This variety has a long history of cultivation and very rich genetic resources, and is an important cash crop in China [6]. The majority of mustard leaves exhibit the presence of trichome. Trichomes are specialized structures that are widely distributed in the above-ground parts of plants, including the leaves, stems, petioles, petals, and glumes [7]. It has been demonstrated that the presence of trichomes is an effective method of deterring insects from feeding on mustard plants, thereby protecting them from damage caused by pests and promoting healthy growth [8]. This is of particular importance for leafy crops, as insect pests not only affect yield, but also reduce quality. Additionally, trichome has been observed to diminish the attachment and invasion of pathogenic bacteria on the leaf surface, consequently reducing the prevalence and dissemination of diseases [9]. The density and morphology of mustard trichome has been demonstrated to fluctuate in response to environmental and temperature variations, which is crucial for their survival and reproduction across diverse geographical regions [10,11].

The existing literature on plant trichome development and regulatory networks has been more thoroughly investigated in the model crop *Arabidopsis thaliana*. In the formation of the MYB-bHLH-WD40 (MBW) complex, the MYB protein initially binds to DNA and recognizes specific promoters of target genes. Subsequently, the basic helix–loop–helix (bHLH) protein interacts with the MYB protein, further stabilizing the complex and augmenting its ability to bind to DNA. The WD40-repeat ( WD40) protein functions as a scaffolding protein, interacting with both the MYB and bHLH proteins to assemble the complete MBW complex [12]. In plants, it regulates the biosynthesis of anthocyanins [13], determines the fate of epidermal cells [14], and influences a variety of other physiological processes [15]. It has been demonstrated that, in *Arabidopsis thaliana*, the R2R3-MYB transcription factor *GLABRA 1* (*AtGL1*) interacts with *GLABRA 3* (*GL3*) and *Transparent Testa Glabra1* (*TTG1*) proteins to form the GL1-GL3-TTG1 trimeric activator complex, and that the MBW complex activates the expression of *GL2*, which induces epidermal trichome development [16]. The MBW complex has been demonstrated to positively regulate trichome development and induce the expression of R3 MYB genes, including *TRICHOMELESS1* (*TCL1*), *TCL2*, *TRYPTICHON* (*TRY*), and others [17]. Given that the simultaneous binding of *GL1* and *GL3* to the promoter of *GL2* via their respective DNA-binding domains may be essential for the activation of *GL2*, these R3 MYB transcription factors can enter neighboring cells to compete with *GL1* in binding to *GL3*. This can inhibit the function of the MBW complex and further regulate epidermal trichome development, representing an alternative approach to epidermal trichome regulation [18].

bHLH is an integral part of the MBW complex formation process and interacts with two other substances that influence each other. This interaction serves to enhance the stability of the complex and to promote its ability to activate downstream genes. Among the known networks of transcriptional regulators, the related bHLH protein *GLABRA3* (*GL3*) and the enhancer of *GLABRA3* (*EGL3*) are thought to have roles in both hairy and non-hairy cells. However, their expression and RNA accumulation preferentially occur in developing hairy cells and are positively regulated by CPC and TRY proteins in *Arabidopsis thaliana* plants [19]. *GL3* is an important component of MBW and is responsible for recognizing and binding to the promoter regions of downstream genes using its own special DNA-binding domains, resulting in the formation of a stable MBW transcriptional activation complex [20]. In tomato plants with the SIMYC1-RNAi construct, type VI glandular hairs display smaller glands and shorter stalks, indicating that SlMYC1 is a crucial regulator of the subsequent stages of type VI glandular hair development in tomato [21]. In addition to its direct involvement in the formation of the MBW complex, the bHLH transcription factor may also regulate this complex through interactions with other signaling pathways that regulate its activity and functions. For example, in poplar, miR319a inhibits the transcriptional activation activity of MBW by specifically targeting and repressing Teosinte branched 1/Cycloidea/Proliferating cell factors (TCP) family genes, which in turn represses the expression of *GL2*, a downstream gene that is a marker of trichome formation and interacts with key factors of the gibberellin (GA) signaling pathway [22].

The basic helix–loop–helix (bHLH) transcription factor(TF) family is one of the largest TF families in eukaryotes [23]. The defining characteristic of the bHLH family is the presence of a DNA-binding bHLH structural domain, which is highly conserved and binds to the E-box sequence (CANNTG), a six-nucleotide sequence present in the promoter sequence. This binding regulates the transcription of target genes [24]. Myelocytomatosis (MYC) is a member of this subfamily, which plays an important role in plant growth and development, and has its own highly conserved HLH domain and a conserved bHLH-MYC-N domain [25]. The bHLH regulators associated with trichome development are *GL3*, *EGL3*, *TT8*, and MYC. The bHLH family of genes is involved in numerous biological processes; a number of reports have been published on genome-wide analyses of bHLH, including studies on *Ziziphus jujuba Mill* [26], *Zea mays* [27], and *Sorghum bicolor* [28]. Studies on the bHLH transcription factor family have identified Chinese cabbage [29], kale [30,31], and oilseed rape [32] to be representative species of cruciferous plants. However, there are few studies related to the mechanism by which the bHLH-MYC complex regulates trichome development in mustard leaves. In this study, we perform a genome-wide identification and expression assay of bHLH-MYC to determine how it regulates the development of trichomes in mustard leaves, including the identification of bHLH-MYC family members and evolutionary and phylogenetic relationships; a series of property analyses, such as the structure and chromosomal distribution of bHLH-MYC genes; and the detection of the amount and the site of expression in trichome/glabrous mustards. The aim of this study was to identify the bHLH-MYC gene, which may be related to the development of leaf trichomes in mustard, and to conduct a preliminary study on its protein structure and function. This study lays a solid foundation for elucidating the mechanisms that regulate the development of leaf trichomes and provides an avenue for the selection and breeding of new varieties. The research results will also provide a valuable theoretical basis for the systematic exploration of the functional properties of mustard trichomes.

## 2. Results

### 2.1. Identification of Gene Family Members and Construction of Evolutionary Tree

**Figure 1:**
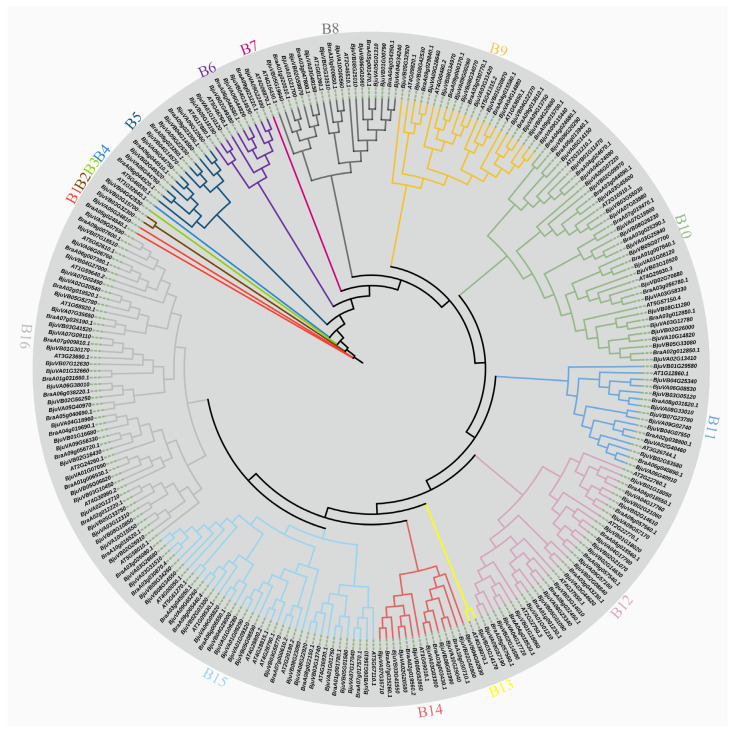
The phylogenetic trees of *Arabidopsis thaliana*, Chinese cabbage, and mustard were constructed using the maximum likelihood method for the binding domains of the bHLH transcription factors. The proteins clustered into 16 distinct groups designated by a group number, B1 to B16, and labeled with different colored branches. Multiple sequence comparisons were performed in accordance with MUSCLE3.8.31 software with the use of default parameters. Phylogenetic trees were constructed using MEGA7, and the results are based on 1000 bootstrap values.

Our comparison and analysis of the Pfam database led to the identification of 40 bHLH family genes in *Arabidopsis thaliana*, 66 in Chinese cabbage (*Brassica rapa*), and 154 in mustard (*Brassica juncea*) (Table S1). In order to explore the evolutionary relationship among them, an unrooted ML tree containing 260 genes was constructed. As illustrated in Figure 1, the tree was divided into 16 groups in accordance with the degree of kinship. The largest groups were B10 (including 37 genes), B12 (33), B15 (38), and B16 (49), which accounted for approximately 60% of the total. In contrast, B3, B4, and B7 each contained only a single member. This suggests that these genes have been relatively conserved during evolution and could provide some reference value for studying the specific functions they play in mustard. Furthermore, this provides a foundation for the construction of a protein interaction network to control the growth of mustard.

### 2.2. Analysis of the Conserved Structural Domains of the Mustard bHLH-MYC Gene Family and the Physicochemical Properties of the Proteins

**Figure 2:**
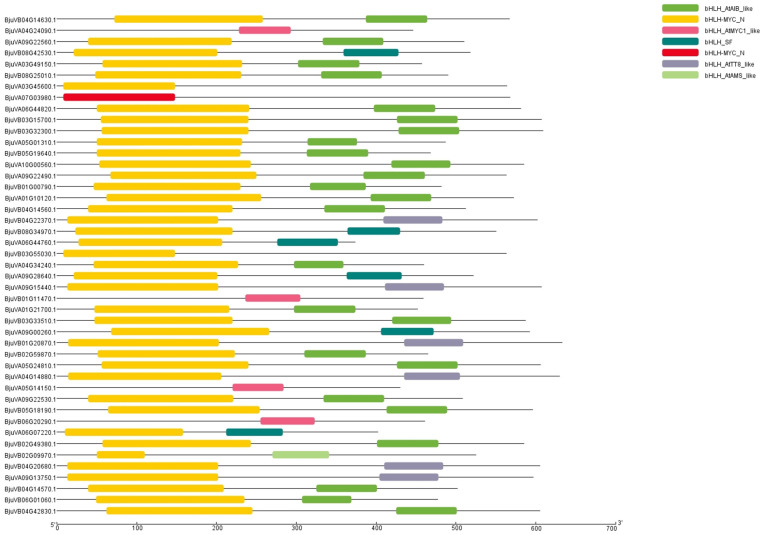
An analysis of the conserved structural domains within the mustard bHLH gene family was conducted. The structural domain information was sourced from NCBI-CDD. Following selection, the final dataset comprised six structural domains, which exhibited comparable amino acid lengths. Among these domains, bHLH-MYC occupied a similar position within the protein sequence.

The binding domain associated with trichome development was found to be bHLH-MYC based on the binding domain analysis of *GL3* in Arabidopsis; the 154 family genes obtained above were screened, and 45 genes associated with bHLH-MYC binding domains were identified (Table S2). Our analysis of the conserved structural domains of mustard bHLH-MYC proteins showed (Figure 2) that the N-terminus of these genes all contained bHLH-MYC-N or bHLH-AtMYC1-like structural domains, and the C-terminus contained the typical bHLH structural domains, including bHLH-AtAIB-Like, bHLH-AtTT8-like, bHLH-AtAMS-like, and bHLH-SF-like domains. It can be appreciated that there is a certain degree of similarity between the family members, but some differences remain. It is possible that these structural domains are involved in different regulatory pathways or are subfamily transcription factors with similar bHLH structural domains. We speculate that the presence of these bHLH structural domains indicates that these genes have a wide range of biological functions and are not just involved in trichome development and the process of regulation; this also implies that we still need to analyze and screen multiple aspects of these family members.

The physicochemical properties of the 45 mustard bHLH-MYC genes were investigated using Expasy online software (https://web.expasy.org/protparam/ accessed on 2 June 2024) (Table 1); the relative molecular masses of the 45 mustard bHLH-MYC genes varied considerably, ranging from 42,633.33 to 70,011.92 kDa. The smallest was *BjuVA06G44760* (transcription factor MYC/MYB N-terminal) and the largest was *BjuVB01G20870* (encodes a basic helix–loop–helix-domain protein that interacts with *GL1* during trichome development). The isoelectric points range from 4.75 to 7.03, with the smallest isoelectric point in *BjuVA04G24090* and the largest isoelectric point in *BjuVA06G44760* (Myc-type, bHLH domain). *BjuVA04G24090*, *BjuVB01G11470*, *BjuVB03G55030*, and *BjuVB03G55030* are acidic proteins, whereas the proteins encoded by *BjuVA06G44760* and *BjuVA01G21700* are nearly neutral.

### 2.3. Chromosome Position Analysis of bHLH-MYC Gene Family Members in Mustard

**Figure 3:**
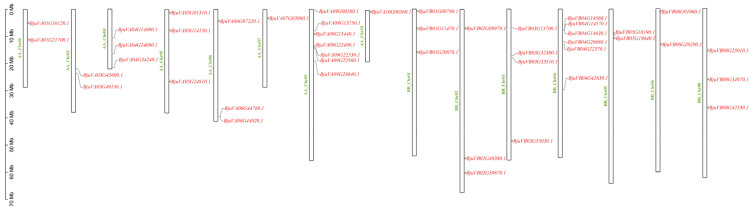
The chromosomal distribution of 45 bHLH-MYC genes in mustard. Chromosome numbers are indicated in green font. Notably, AA_Chr02, AA_Chr08, and BB_Chr07 do not harbor any of these genes, while 15 chromosomes are depicted in the figure as carrying them. The bHLH-MYC family members are denoted in red font. Specifically, AA_Chr09 contains 7 members, AA_Chr07 and AA_Chr10 each have 1 member, and the remaining members are more evenly distributed across the other chromosomes.

Gene structure annotation information from the mustard genome was employed to determine the chromosomal physical location of each gene. These data were then visualized and analyzed using TBtools v2.136 software. The results showed that AA_Chr02, AA_Chr08, and BB_Chr07 are absent, while the other bHLH-MYC members are present on 15 chromosomes. Notably, chromosome AA_Chr09 has the highest number of bHLH-MYC genes, with a total of seven. In addition, the AA_Chr 07 and AA_Chr 10 chromosomes each contained one bHLH-MYC family gene (Figure 3).

### 2.4. A Conservative Analysis of the Gene Sequences and Gene Structures of the Mustard bHLH-MYC Gene Family Members

**Figure 4:**
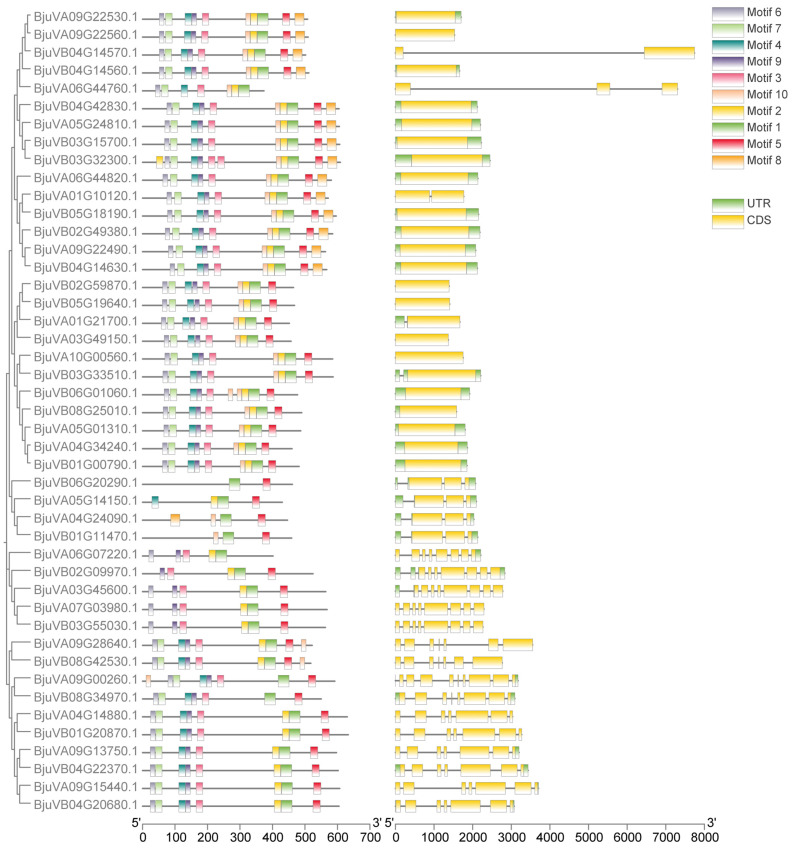
Conservation analysis of 45 bHLH-MYC family genes, including cluster analysis, amino acid sequence motif analysis, and exon and intron evaluation. Motif number is defined as 10, and other parameters are specified as default.

In order to further explore the diversity and gene structure of the mustard bHLH-MYC subfamily genes, we performed a motif analysis of 45 mustard bHLH-MYC amino acid sequences using the MEME program (Figure 4), and a total of 10 motifs were identified. The conserved motif analysis map demonstrates that the protein molecules encoded by the mustard bHLH-MYC subfamily genes contain between 2 and 10 motifs. Of the 45 genes, 1 (*BjuVB06G20290*) has 2 motifs, 15 have 10 motifs, and the remaining 29 have between 2 and 9 motifs. This indicates that the gene is relatively conserved, and that 45 of the mustard bHLH-MYC subfamily members contain motifs 1 and 5. The presence of conserved motifs indicates that these two motifs are the most conserved. TBtools v2.136 software was employed to analyze and identify the exons and introns of the bHLH-MYC family genes (Figure 4), and showed that all genes contained more than one exon. The number of introns exhibited a polarized distribution, with some genes containing no introns and others containing multiple. Among these, 21 genes had no introns, while 15 genes had multiple introns. Notably, the gene with the largest number of exons was *BjuVB09G00260*, which had nine exons. This suggests that the gene structure of the mustard bHLH-MYC gene family is relatively simple and that the family exhibits a certain degree of conservation.

### 2.5. Co-Linearity Analysis of the Mustard bHLH-MYC Gene Family

**Figure 5:**
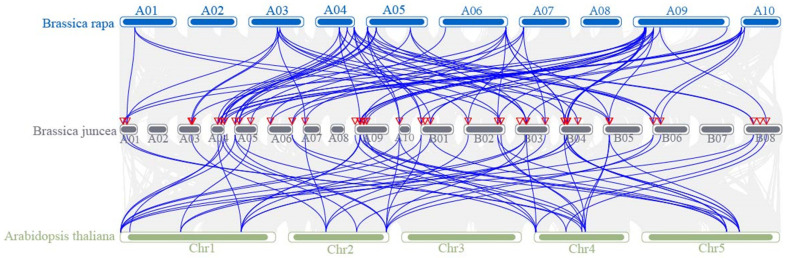
The collinearity analysis of bHLH-MYC family genes between *B. rapa*, *B. juncea*, and *A. thaliana*. A01–A10 represent 10 chromosomes in *B. rapa*, A01–B08 represent 18 chromosomes in *B. rapa*, and Chr1 to Chr5 represent 5 chromosomes in *A. thaliana*.

In order to ascertain the degree of conservation of this family during the evolutionary process, it was analyzed for interspecific (*Arabidopsis thaliana*, Chinese cabbage, mustard) and intraspecific covariance using MCScanX function in TBtools v2.136. The interspecific covariance analysis (Figure 5) demonstrated that the family genes were absent from chromosomes A02 and A08. Furthermore, chromosome B07 for mustard corresponds to A02 and A08 for Chinese cabbage and Chr3 for *Arabidopsis*. The bHLH-MYC genes of mustard exhibited 70 pairs of covariance with cabbage and 55 pairs of covariance with *Arabidopsis*. The covariance between mustard and cabbage is greater than that between mustard and *Arabidopsis thaliana*, which may be a result of the fusion of mustard with the cabbage AA genome. This also suggests that the bHLH gene family is highly conserved in mustard during the evolutionary process.

**Figure 6:**
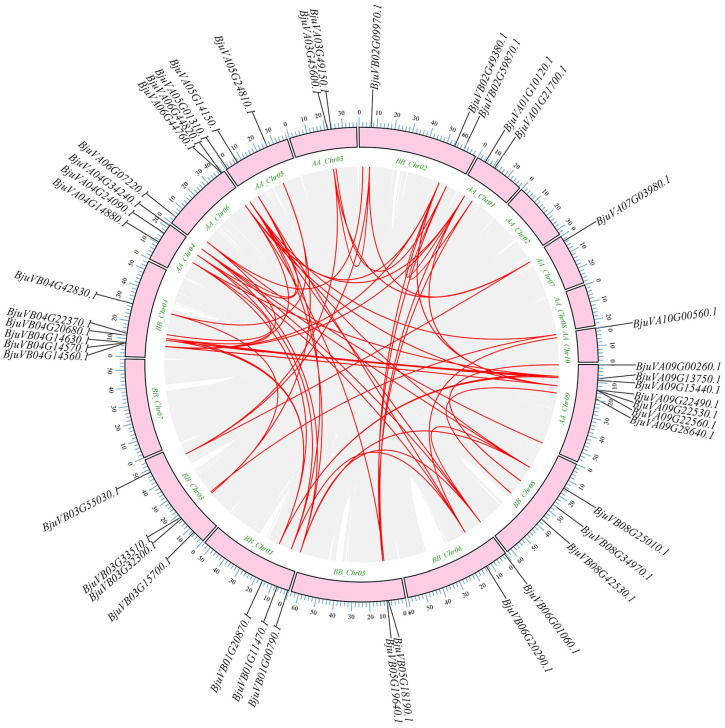
The collinearity analysis of mustard bHLH-MYC genes. The red line indicates the linear correlation of genes in the mustard bHLH-MYC family. More red lines for the same gene show highly conserved homology.

Meanwhile, the intraspecific covariance analysis plot (Figure 6) indicates that 15% of the genes have few homologous gene pairs, while the majority have multiple homologous gene pairs. This further substantiates our hypothesis that the genes under consideration are highly homologous to each other, suggesting that they share a similar structure or perform analogous functions.

### 2.6. Expression Analysis of bHLH-MYC Family Genes on Leaf Trichome

**Figure 7:**
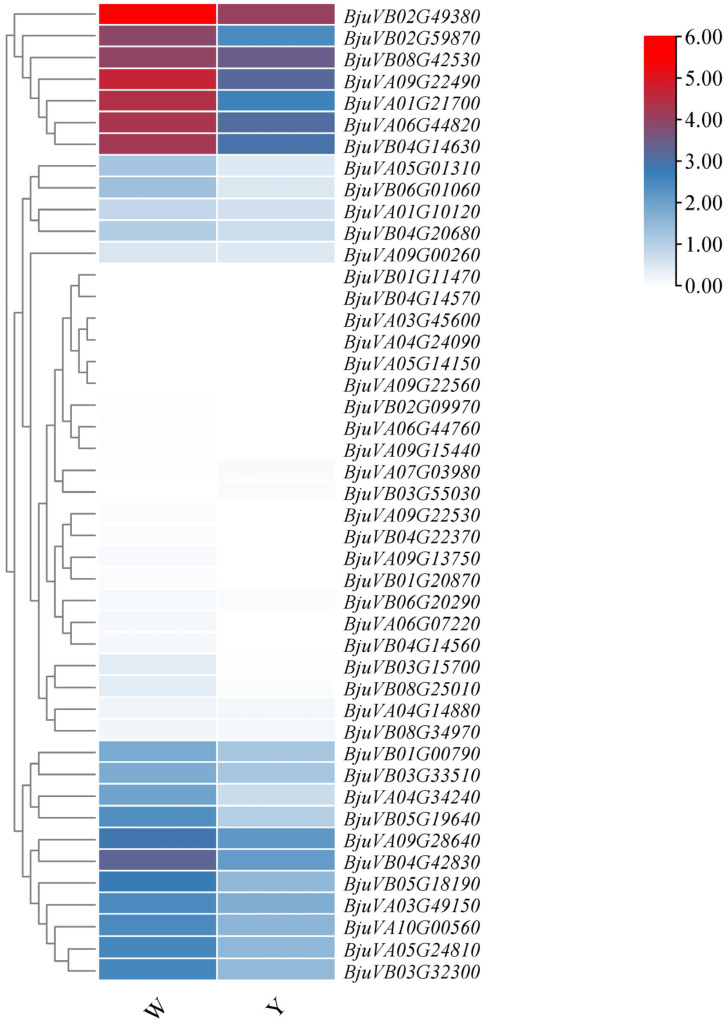
The expression levels of the 45 bHLH-MYC family members. Material denoted as w possesses trichome, whereas material denoted as y is glabrous. Significant differences in the expression of these family members were observed between the two types of material. Following selection, *BjuVB04G14560*, *BjuVA05G24810*, *BjuVA06G44820*, *BjuVA09G22490*, and *BjuVA09G13750* were identified as being potentially associated with trichome development.

To better understand the expression patterns of bHLH-MYC family genes in Y (glabrous material) and W (trichome material), we used adult leaves from these two materials as test samples. The expression results of the bHLH-MYC family genes after Log processing were summarized using a heat map, as depicted in Figure 7. Notably, six genes (*BjuVA03G45600*, *BjuVA04G24090*, *BjuVA05G14150*, *BjuVA09G22560*, *BjuVB01G11470*, and *BjuVB04G14150*) were not expressed in either Y and W. Additionally, nine genes showed no expression in Y, while one gene (*BjuVB03G55030*) demonstrated no expression in W. Compared to W, only two genes, namely *BjuVA07G03980* and *BjuVB03G55030*, exhibited up-regulation, with the remaining genes showing down-regulation. Based on the gene annotation results, four MYC-related genes (*BjuVB04G14560*, *BjuVA05G24810*, *BjuVA06G44820*, and *BjuVA09G22490*) and one *GL3*-related gene (*BjuVA09G13750*) were identified. The expression of these five genes was up-regulated in W. It has been reported that MYC transcription factors may indirectly influence trichome formation through the formation of complexes or by interacting with other transcription factors that regulate this process (such as the TCP transcription factors and MYB transcription factors, etc.). For instance, TCP transcription factors have been shown to directly inhibit the activity of the MYB-bHLH-WD40 (MBW) complex, which is crucial for regulating trichome formation [33]. In contrast, MYC transcription factors may exert indirect control over the functionality of the MBW complex by influencing the expression or activity of TCP or other related transcription factors [34]. The precise genes that are directly regulated by MYC are not yet fully understood. However, MYC transcription factors are generally recognized to function as transcriptional activators, binding to the promoter regions of target genes and thereby promoting their expression. This confirms that MYC transcription factors may influence trichome formation by up- or down-regulating the expression of genes associated with this process.

### 2.7. Three-Dimensional Structure Prediction, Sequence Logo Analysis, and Subcellular Localization of bHLH-MYC Transcription Factors

**Figure 8:**
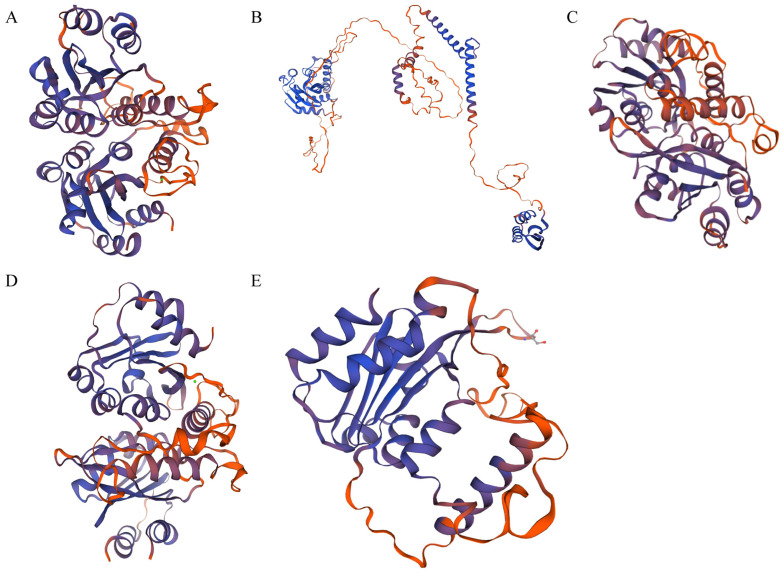
Three-dimensional (3D) structure analyses of the bHLH-MYC protein ((**A**–**E**) depict the protein structures of mustard bHLH-MYC, corresponding to the genes *BjuVA09G22490*, *BjuVA09G13750*, *BjuVB04G14560*, *BjuVA05G24810* and *BjuVA06G44820*).

To investigate the functional implications of protein structures, we utilized SWISS-MODEL to generate three-dimensional (3D) structural models for the aforementioned five genes, which exhibited highly divergent protein 3D architectures. Consequently, our analysis was specifically directed towards the characteristics of the MYC N-terminal during the modeling phase. As illustrated in Figure 8, these five proteins exhibit both similarities and differences in their 3D structures. Specifically, the protein *BjuVA05G24810* contains 7 α-helices, *BjuVB04G14560* has 9 α-helices, *BjuVA09G22490* possesses 12 α-helices, *BjuVA06G44820* features 8 α-helices, and *BjuVA09G13750* comprises 15 α-helices and 10 β-sheets. Notably, four of these proteins share 6 β-folded regions, with the exception of *BjuVA09G13750*, which harbors 10 β-folded regions. The inclusion of 10 β-folded regions in *BjuVA09G13750* may justify the selection of *GL3* as a reference in the modeling process, given its dual similarity to MYC in structure and its involvement in additional functional roles. The amino acid residues situated on the surface of the β-folds are capable of specifically recognizing and binding to the corresponding structural domains of other proteins. The number and positional conservation of these residues may provide insights into their potential role in subsequent transcriptional regulatory processes. Furthermore, this observation allows us to hypothesize that the β-folded region may be the functional domain through which bHLH-MYC exerts its effects.

**Figure 9:**
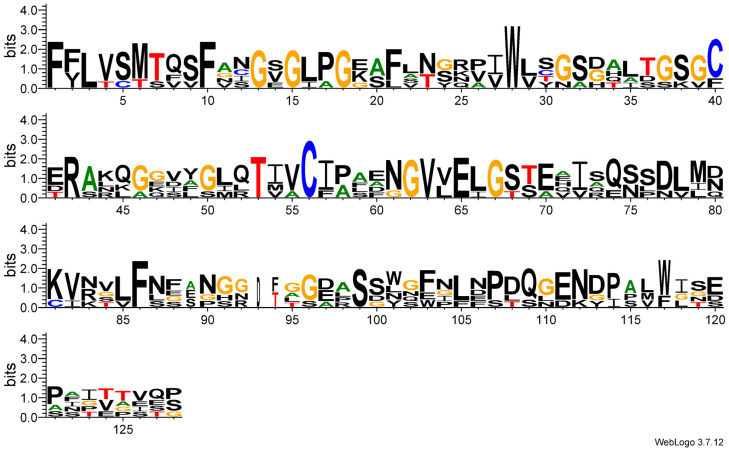
The sequence logo analyses of the bHLH-MYC of mustard. In the sequence logo analysis images, the height of the letter indicates how often that amino acid occurs at that position, implying that the type of amino acid at that position is relatively fixed in the set of sequences being analyzed and may be important for the function or structure of the sequence.

In addition to their three-dimensional (3D) structure, the sequence signatures of the proteins highly associated with trichome development through bHLH-MYC were analyzed (Figure 9). Using GL3 as the reference sequence and comparing sequences in Jalview, WebLogo 3’s sequence logo analysis revealed that its amino acid sequence was highly conserved during evolution. The three-dimensional structure analysis shows that the protein structures of these genes are highly similar. The relatively conserved amino acids include phenylalanine, serine, threonine, cysteine, and arginine, which are crucial for the three-dimensional structure and function of the protein. A significant amino acid bias was present at 21 sites, including 4, 5, and 40. These amino acids were not selected to produce an amino acid bias, while some other amino acids were selected as key functional amino acids. Analyzing the sequence signatures of bHLH-MYC will facilitate the future identification of key functional amino acids.

**Figure 10:**
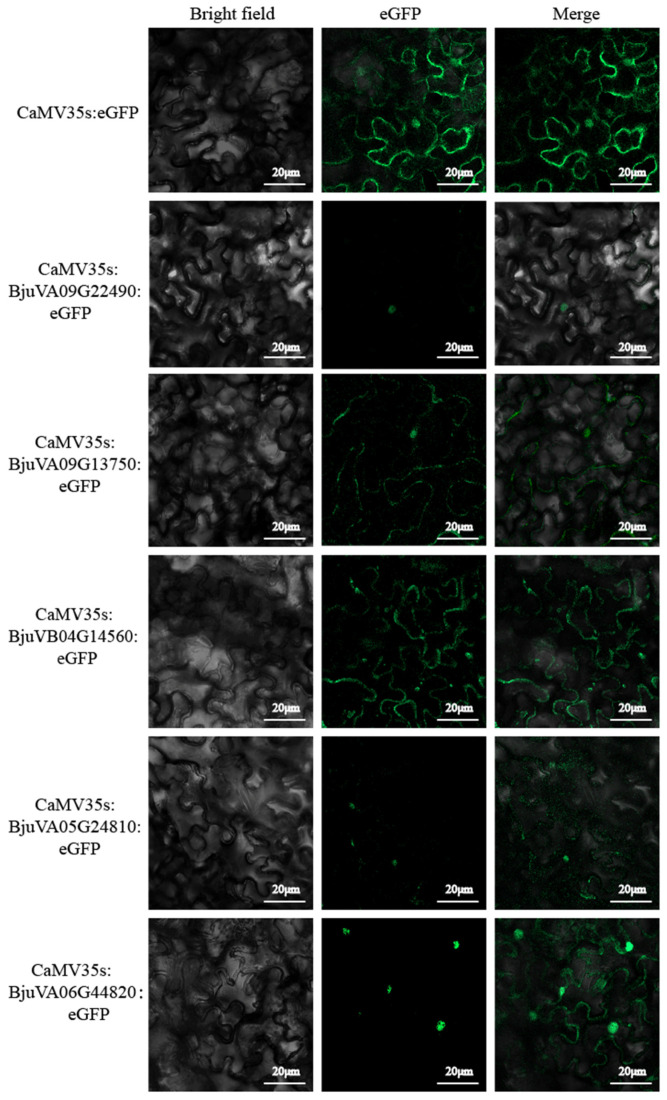
Potential functions of mustard bHLH-MYC. Subcellular localization of five mustard bHLH-MYCs that were transiently expressed in *N. benthamiana* leaves using eGFP as a control. Bright field indicates bright field microscopy image, eGFP indicates green fluorescence image, and merge indicates merged bright field and green fluorescence image.

In order to elucidate the mechanism of action of MYC transcription factors, it was essential to ascertain the site of action of their expression products. The WoLF PSORT tool (WoLF PSORT: The Advanced Protein Subcellular Localization Prediction Tool—GenScript) and Cell-PLoc (Cell-PLoc 2.0 package, www.sjtu.edu.cn (accessed on 30 September 2024)) were employed for this purpose. The predictions of *BjuVA09G22490, BjuVA09G13750, BjuVB04G14560, BjuVA05G24810,* and *BjuVA06G44820* were conducted concurrently. Our predictions indicate that the proteins encoded by these bHLH-MYC genes are expressed in the nucleus. The constructed pCAMBIA2300 vector with the eGFP fluorescent tag was transferred into Agrobacterium to infect *N. benthamiana*. Following our observation of the expression sites of the target genes under a confocal microscope (Figure 10), it was found that their encoded proteins were expressed in the nucleus, where they realized expression regulatory functions. The actual results are consistent with the predicted results, which may be helpful for further verification of the specific functions of target genes in the nucleus.

## 3. Discussion

### 3.1. The MBW Ternary Complex Plays a Pivotal Role in Regulating the Characteristics of Mustard Trichomes

The MBW ternary complex is constituted by three transcription factors, namely the R2R3MYB, bHLH, and WD40 proteins. It induces trichome development by directly regulating the expression of downstream genes, including *GL2* [35]. *GL2* is a key gene that controls the development of epidermal trichomes, which is tightly regulated by the MBW complex. In addition to promoting the differentiation of epidermal trichomes, the MBW complex also responds to changes in external factors, such as the effects of other transcription factors and environmental stimuli, to regulate its activity. These regulatory mechanisms collectively determine the number and density distribution of mustard trichomes [36]. Therefore, the MBW ternary complex is pivotal in the regulation of trichome traits in mustard and other plants. An understanding of its structural composition and molecular regulatory network will prove invaluable for studying the molecular mechanisms of plant epidermal trichomes; in this paper, we focused on the effects of the bHLH transcription factor, a component of the MBW ternary complex, on the development of mustard trichomes.

In recent years, genome analyses and the cloning of bHLH-MYC-related members in various species have shown that bHLH TFs may be regulated by post-translational modifications (PTMs) involving the homo- or heterodimerization of their bHLH structural domains. In addition, it has been proposed that a range of PTMs may play important roles in regulating subcellular localization, DNA binding capacity, transcriptional activity, and stability (e.g., protein–protein interactions, phosphorylation, ubiquitination) [37]. MYC is a member of the bHLH subfamily of transcription factors and is involved in the regulation of the citrus flavonoid biosynthesis pathway, in addition to its association with the formation of trichomes [38]. MYC2 is also a member of the family and is a key factor involved in the jasmonate response pathway, which is important in strawberry resistance to stresses such as low temperatures, drought, and salt stress [39]. It is clear that bHLH-MYC plays a key role in many biological processes in plants, including trichome development, jasmonic acid signaling, anthocyanin biosynthesis, the perception of light-sensitive pigment signals, and responses to adversity stress [40]. These studies suggest that bHLH is involved in the regulatory process of trichome development and in the regulatory pathways of other biological processes. However, the regulatory process of bHLH-MYC in the development of Chinese mustard leaf trichomes has not yet been investigated. Therefore, in this study, we carried out preliminary research into bHLH-MYC’s role in the development of mustard trichomes. In this study, we identified bHLH-MYC transcription factors and selected mustards with and without trichomes for transcriptome analysis. Our findings indicate that the expression of these transcription factors is significantly elevated in mustard with trichomes compared to those without. In addition, we observed instances of non-expression, which could be attributed to base deletions leading to the premature termination of translation. Combined with the finding of subcellular localization, all five genes were found to be expressed in the nucleus. This suggests that the function of these transcription factors may be related to the regulation of trichomes in multiple ways.

### 3.2. bHLH Is an Integral Part of the MBW Complex and Is Important for Mustard Trichome Development

The bHLH family is one of the largest families of transcription factors [41]. It is widely present in plants and animals and is involved in numerous biological processes, such as development, photosynthesis, anti-adversity responses, and secondary metabolism [42,43]. Together with MYB and WD40, it forms the MBW complex, which regulates the transcriptional activity of downstream target genes by specifically binding to the promoter region, thus controlling trichome development. In *Arabidopsis thaliana*, a great deal of research has been conducted on the MBW complex [44], finding that trichome development is mainly regulated by positive regulators such as R2R3-MYB, WD40 (WD40-repeat), bHLH (basic helix–loop–helix), and C2H2 (C2H2 zinc finger protein), as well as R3-MYB-like negative regulators [45]. The positive regulator bHLH possesses many family members which have different modes of regulation and functions, thus playing roles in different plants. For example, *GL3*, *EGL3*, *TT8*, and *MYC1* [46] are encoded bHLH-type positive transcription factors. This shows that bHLH proteins are important components of the MBW complex in terms of the regulation of trichomes. In this study, we focused on the effect of bHLH-MYC on trichome development and screened five genes associated with trichome development. Four of them were associated with the positive transcription factor MYC, and one gene was associated with the transcription factor *GL3*, which is consistent with the results of previous studies. We will continue to carry out further validation of the functions of these genes, as well as exploring their reciprocal regulatory networks.

### 3.3. Future Research Directions for Mustard Trichomes

Mustard trichomes are of significant biological importance as they allow the plant to adapt to changes in the environment and confer resistance to pests and diseases. However, there is a general preference for glabrous vegetables, and the presence of trichomes affects the sensory experience and visual appearance of vegetables. Therefore, it is important to understand the regulatory mechanisms of trichome development and to utilize modern molecular techniques, such as CRISPR, VIGS, and transgenics, to alter the formation of trichomes; as a significant future research area for mustard trichomes, molecular markers can be employed in the breeding process for selective breeding.

## 4. Materials and Methods

### 4.1. Plant Materials

The mustard material was obtained from the Cabbage Group of the Institute of Vegetable and Flower Research, Chinese Academy of Agricultural Sciences. The glabrous material, obtained from the hybridization of *Brassica juncea* var. *capitata* and root mustard, was labeled Y, while the trichome material from all-purpose mustard was labeled W. Both materials were high-generation self-crosses. They were planted in the open air and sampled after one month. The sampling site was the third true leaf from the heart leaf outwards. Three replicates of each material were taken and placed in liquid nitrogen and stored at −80 °C for RNA extraction and RNA sequencing analysis.

### 4.2. Identification of the Members of the Mustard bHLH-MYC Gene Superfamily and Construction of an Evolutionary Tree

The genome sequences of mustard (T84-66), Chinese cabbage, and *Arabidopsis thaliana*, along with the relevant annotation files, were obtained. The data were obtained from BRAD (http://39.100.233.196:82/download_genome/Brassica_Genome_data/Braju_tum_V2.0/ accessed on 19 May 2024). A total of 161 AtbHLH protein sequences were downloaded from TAIR (www.arabidopsis.org accessed on 19 May 2024) and used as queries for BLASTP searches within the local protein database of mustard [47]. The HMM files for the conserved structural domains of bHLH (PF14215 and PF00010) were downloaded from the Pfam database (http://pfam.xfam.org/ accessed on 19 May 2024) [48]. The tumida self-cross T84-66 was mapped through the HMM corresponding to the binding domain (PF14215), with the threshold set to an E-value of less than 1 × 10^−10^. The putative mustard bHLH and the putative *Brassica rapa* bHLH were then obtained using a combination of the HUMMER and BLASTP methods. Finally, the functional structural domains were screened using hmmsearch (hmmsearch search|HMMER (www.ebi.ac.uk accessed on 22 May 2024)), and MUSCLE software with preset parameters was utilized for a multiple-sequence comparison of the protein sequences of the identified bHLH family genes in *Arabidopsis thaliana*, cabbage, and mustard. A maximum likelihood tree was constructed using MEGA X10.2.6 software based on the Jones–Taylor–Thornton (JTT) model with a bootstrap test of 1000 replicates, based on the results of the multiple-sequence alignment. The phylogenetic tree was visualized in MEGAX [49] and retouched using the ITOL online webpage (iTOL: Interactive Tree Of Life (https://itol.embl.de/ accessed on 27 May 2024)) [50].

### 4.3. Analysis of Conserved Structural Domains of the Mustard bHLH-MYC Gene Family and Physicochemical Properties of Proteins

The protein files of the genes mentioned above were uploaded to the NCBI-CDD server (https://www.ncbi.nlm.nih.gov/Structure/cdd/wrpsb.cgi accessed on 23 May 2024) and visualized using the Visualize NCBI CDD Domain Pattern function of TBtools v2.136 [51]. The Expasy website (https://web.expasy.org/protparam/ accessed on 2 June 2024) was utilized to conduct analyses of the isoelectric point and the relative molecular mass of the mustard bHLH proteins [52].

### 4.4. Chromosomal Localization of Mustard bHLH-MYC Gene Family Members

By employing the ‘Amazing Gene Location From Gff3 File’ function of TBtools v2.136 software [51], in conjunction with the annotation data pertaining to the mustard gene structure and the IDs of the mustard bHLH-MYC gene family members, we ascertained the distribution of the mustard bHLH-MYC gene family members on the chromosomes and subsequently constructed a chromosomal localization map of the mustard bHLH-MYC gene family.

### 4.5. Gene Structure Analysis of the Mustard bHLH-MYC Gene Family

The Gene Structure Show function of TBtools v2.136 software [51] enabled the distribution of the introns, exons, and non-coding regions of the genes to be obtained by combining the annotation information of the mustard gene structures and the IDs of the mustard bHLH gene family members.

### 4.6. Analysis of Conserved Motifs of the Mustard bHLH-MYC Protein and Phylogenetic Tree Analysis

The protein sequences encoded by members of the mustard bHLH gene family were analyzed using the MEME website (https://meme-suite.org/ accessed on 5 June 2024) [53], with the number of motifs set to 10 and the other parameters set to default values [54,55]. The sequences were then analyzed using TBtools v2.136 software [51] to map the conserved motif analysis. The Muscle software was employed to facilitate a comparative analysis of the mustard bHLH gene sequences, with the construction of a phylogenetic tree undertaken using the maximum likelihood method. This was subsequently modified using Figtree v1.4.4 software.

### 4.7. Covariance Analysis of the Mustard bHLH-MYC Gene Family

The genes within this family were subjected to an analysis of interspecific and intraspecific covariance using the MCScanX function in TBtools v2.136 software [56].

### 4.8. RNA-Seq Analysis

Total RNA was extracted from the fresh leaves of the glabrous material and trichome material using TRIzol^®^ Reagent according to the manufacturer’s instructions (Invitrogen, Carlsbad, CA, USA), and genomic DNA was removed using DNase I (Tokyo, Japan, TaKara). Then, RNA quality was determined using a 2100 Bioanalyser (Agilent, Santa Clara, CA, USA) and quantified using the NanoDrop 2000 Sepectrophotometer (Thermo, Wilmington, NC, USA) [57]. After quantification with TBS380, paired-end libraries were sequenced using Illumina NovaSeq 6000 sequencing (150 bp*2, Shanghai BIOZERON Co., Ltd., Shanghai, China).

### 4.9. Three-Dimensional (3D) Structure and Sequence Identity Analysis of the bHLH Protein

The five selected bHLH genes were analyzed for protein 3D structure prediction using the online website SWISS-Model (SWISS-MODEL (www.expasy.org)) [58]. In addition, their amino acid sequences were aligned, and the sequence signatures of the mustard bHLH family of genes were established for identity analysis using WEbLOGO3 (WebLogo 3-About (www.threeplusone.com accessed on 10 September 2024)) [59].

### 4.10. Subcellular Localization

#### 4.10.1. Subcellular Localization Prediction

Subcellular localization prediction was conducted by utilizing the Cell-PLoc2.0 tool (http://www.csbio.sjtu.edu.cn/bioinf/Cell-PLoc-2/ accessed on 13 September 2024) [60] and the WOLF PSORTII programs (https://wolfpsort.hgc.jp/ (accessed on 13 September 2024) [61].

#### 4.10.2. Vector Construction of Subcellular Localization

The sequence information necessary for gene cloning was obtained from BRAD (http://brassicadb.org/brad/ accessed on 13 August 2024). The amplification protocol involved pre-denaturation at 95 °C for 3 min, followed by 35 cycles of denaturation at 95 °C for 30 s, annealing at 57 °C for 30 s, and an extension at 72 °C for 60 s, with a final extension step at 72 °C for 10 min. The amplified sequences were verified through sequence alignment using DNAMAN 8.0 software. The pCAMIBA2300 plasmid was double-digested with BamHI and XbaI enzymes. The primers for cloning and recombination were designed utilizing Oligo7 software (Table S3). Vector construction was carried out with a homologous recombination kit [62].

#### 4.10.3. Cultivation and Infestation of *N. benthamiana*

1. *N. benthamiana*, originating from Australia, was propagated in trays containing sterilized peat soil and subsequently incubated in a constant-temperature incubator until the plants reached the 5–6-leaf stage, at which point infestation occurred.
2. Utilizing the vector derived from strain GV3101 as described in Section 4.10.2, the target gene was expressed in fusion with a reporter gene (eGFP). The subcellular localization of the target gene was then determined by observing the expression pattern of the reporter gene [63].

## 5. Conclusions

This study represents the first comprehensive and systematic study of mustard bHLH-MYC and trichome development. A total of 45 bHLH-MYC members were identified which possessed molecular biological features, such as relatively consistent gene structures and conserved structural domains. Transcriptome analysis, subcellular localization, and protein structure analysis were performed on five genes that might be related to the development of mustard trichomes. The expression levels of these five genes were significantly elevated in the trichome material (W) in comparison to the glabrous (Y) material. This demonstrates the importance of bHLH-MYC in trichome development, which is helpful for subsequent studies of the interactions between bHLH family proteins, signaling pathway regulation, and its function in trichome development. Meanwhile, the results of the present study provide some basic resources for studying the function of bHLH transcription factors in mustard, promoting the improvement of mustard’s quality and economic benefits.

## Figures and Tables

**Table 1:** Analysis of physicochemical properties of proteins of the mustard bHLH gene family.

Gene	PI	MW/kDa	Gene	PI	MW/kDa
*BjuVA01G10120*	5.30	62,473.40	*BjuVB01G11470*	4.92	52,058.65
*BjuVA01G21700*	6.69	50,298.94	*BjuVB01G20870*	5.99	70,011.92
*BjuVA03G45600*	5.15	63,848.04	*BjuVB02G09970*	5.24	59,529.26
*BjuVA03G49150*	6.14	51,060.68	*BjuVB02G49380*	5.14	64,050.93
*BjuVA04G14880*	6.14	69,968.95	*BjuVB02G59870*	6.20	51,924.52
*BjuVA04G24090*	4.75	50,446.84	*BjuVB03G15700*	5.32	65,953.75
*BjuVA04G34240*	5.34	51,262.59	*BjuVB03G32300*	5.22	65,999.69
*BjuVA05G01310*	5.44	54,312.47	*BjuVB03G33510*	6.24	65,008.89
*BjuVA05G14150*	5.45	48,266.04	*BjuVB03G55030*	4.97	63,948.32
*BjuVA05G24810*	5.21	65,777.46	*BjuVB04G14560*	5.37	56,625.21
*BjuVA06G07220*	5.72	46,181.86	*BjuVB04G14570*	5.61	55,457.99
*BjuVA06G44760*	7.03	42,633.33	*BjuVB04G14630*	5.19	62,049.94
*BjuVA06G44820*	5.08	63,478.29	*BjuVB04G20680*	5.48	67,676.33
*BjuVA07G03980*	5.02	64,364.66	*BjuVB04G22370*	5.54	67,082.36
*BjuVA09G00260*	5.82	66,992.63	*BjuVB04G42830*	5.31	65,717.57
*BjuVA09G13750*	5.47	66,340.49	*BjuVB05G18190*	5.02	65,125.24
*BjuVA09G15440*	5.23	67,879.28	*BjuVB05G19640*	6.02	52,633.44
*BjuVA09G22490*	5.05	61,843.65	*BjuVB06G01060*	5.57	53,093.87
*BjuVA09G22530*	5.64	56,259.17	*BjuVB06G20290*	5.40	51,484.77
*BjuVA09G22560*	5.50	56,312.12	*BjuVB08G25010*	5.80	54,713.74
*BjuVA09G28640*	5.50	59,989.01	*BjuVB08G34970*	5.65	62,234.17
*BjuVA10G00560*	6.06	64,164.85	*BjuVB08G42530*	5.90	59,225.44
*BjuVB01G00790*	5.33	53,306.82			

## Data Availability

The data presented in this study are openly available in the NCBI with the BioProject number PRJNA1187872. (https://www.ncbi.nlm.nih.gov/search/all/?term=PRJNA1187872, accessed on 24 June 2024).

## Supplementary Materials

The following supporting information can be downloaded at https://www.mdpi.com/article/10.3390/plants14020268/s1, Table S1: Members of the bHLH family in each of the three species; Table S2: Family members with bHLH-MYC structural domains; Table S3: Primer sequence information for members of bHLH-MYC.
